# Finding Similarities in Differences Between Autistic Adults: Two Replicated Subgroups

**DOI:** 10.1007/s10803-023-06042-2

**Published:** 2023-07-12

**Authors:** Tulsi A. Radhoe, Joost A. Agelink van Rentergem, Carolien Torenvliet, Annabeth P. Groenman, Wikke J. van der Putten, Hilde M. Geurts

**Affiliations:** 1 Brain & Cognition, Department of Psychology, Dutch Autism & ADHD Research Center (d’Arc), University of Amsterdam, Nieuwe Achtergracht 129-B, 1018 WS Amsterdam, The Netherlands; 2 Research Institute for Child Development and Education, University of Amsterdam, Nieuwe Achtergracht 129-B, 1018 WS Amsterdam, The Netherlands; 3 Leo Kannerhuis (Youz/Parnassiagroep), Overschiestraat 57, 1062 HN Amsterdam, The Netherlands

**Keywords:** Autism, Individual variability, Heterogeneity, Subtype, Validity, Aging

## Abstract

Autism is heterogeneous, which complicates providing tailored support and future prospects. We aim to identify subgroups in autistic adults with average to high intelligence, to clarify if certain subgroups might need support. We included 14 questionnaire variables related to aging and/or autism (e.g., demographic, psychological, and lifestyle). Community detection analysis was used for subgroup identification in an original sample of 114 autistic adults with an adulthood diagnosis (autism) and 58 non-autistic adults as comparison group (COMP), and a replication sample (*N*_Autism_ = 261; *N*_COM*P*_ = 287), both aged 30–89 years. Next, we identified subgroups and assessed external validity (for cognitive and psychological difficulties, and quality of life [QoL]) in the autism samples. To test specificity, we repeated the analysis after adding 123 adults with ADHD, aged 30–80 years. As expected, the autism and COMP groups formed distinct subgroups. Among autistic adults, we identified three subgroups of which two were replicated. One of these subgroups seemed most vulnerable on the cluster variables; this subgroup also reported the most cognitive and psychological difficulties, and lowest QoL. Adding the ADHD group did not alter results. Within autistic adults, one subgroup could especially benefit from support and specialized care, although this must be tested in future studies.

**Supplementary Information:**

The online version contains supplementary material available at 10.1007/s10803-023-06042-2.

The search for support for people with psychiatric conditions is complicated by interindividual differences in key characteristics, support needs, and prognosis, even between people with the same diagnostic classification. Characteristics of a diagnostic category are not necessarily caused by one specific mechanism (Klahr et al., 2012), and several different combinations of mechanisms may cause the same characteristics (equifinality). Whether differences between people within a diagnostic category are so large that it becomes necessary to establish subgroups for whom different variables are central to experienced challenges and solutions has been debated for decades (Buchanan & Carpenter, 1994; Feczko et al., 2019; Lombardo et al., 2019; Wardenaar & de Jonge, 2013). However, while the overarching category can make sense and subclassifications are not needed for diagnostic purposes, it could be relevant to look for subgroups within a classification to provide better support and more insight in a specific individual’s prognosis. In the current study, we aim to test whether we can identify such subgroups within the autism spectrum to increase our insight into this heterogeneity to better inform autistic adults and clinicians what being autistic could entail for them. Thus, the goal is not to develop new diagnostic categories or to aid the search for a cause of a specific classification, but rather to focus on subgroup identification within an existing diagnostic category that can increase the likelihood of receiving proper support. To reach this goal, we test our results using several validation approaches.

Heterogeneity across those diagnosed with an Autism Spectrum Disorder (ASD) (American Psychiatric Association, 2013) has been widely acknowledged, as large differences exist in the depth, presentation, and causes for the diverse characteristics that define autism (Happé et al., 2006; Masi et al., 2017; Mottron & Bzdok, 2020). Currently, a broad diagnostic label for autism is used in the DSM-5 (American Psychiatric Association, 2013), i.e., Autism Spectrum Disorder, that acknowledges interindividual differences and is more inclusive (Frith & Happé, 2020). The downsides of this broad classification are the difficulty to inform people about the specific prognosis, and there is less guidance in which characteristics are most amenable for providing support.

There have been previous studies indicating that subgroups in autism samples can be found, for example in brain structures (H. Chen et al., 2019), inflammation markers (Sacco et al., 2012), and electroencephalography (EEG) (DiStefano et al., 2019). Most of these studies have a fundamentally different goal than the current subgrouping study. These studies inform us about biological differences and are designed to inform about causes and markers. In the long run this might be informative for prognosis, but these studies do not necessarily lead to clinical insights that are directly relevant to support or prognosis. Moreover, previous subgrouping research is often not informative for autistic adults, because studies (a) mostly focus on children or adolescents, (b) often include small sample sizes, (c) focus on a single outcome, (d) are cross-sectional, and (e) adopt few or no validation or replication techniques. A recent review on subgrouping research in autism indicates that few studies are explicit about when the observed subgroups are considered valid, replicate results in a second sample, or investigate stability of subgroup membership over time (Agelink van Rentergem et al., 2021). Moreover, of the 156 studies included in our review, the majority (89%) focused on children or adolescents, showing a clear need for more research on autistic adults.

Of these studies included in the review, we would like to highlight two studies that identified clinically relevant subgroups in autistic adults. One study identified three subgroups in 180 autistic adults aged 23–60 years using normative outcomes and indicators of quality of life (Bishop-Fitzpatrick et al., 2016). These subgroups differed in their level of dependence (e.g., living independently, semi-independently, or with full-time oversight by staff and/or family), employment status, and physical/mental health. In a different study, five groups were identified in 55 adults with autism and ADHD (mean age 34 years) that differed in employment status, educational level attained, age at diagnosis and need of hospitalization (LaBianca et al., 2018). Although these studies were conducted for different goals, they show that homogeneous, subgroups can be identified in autistic adults that are potentially clinically meaningful. However, the replicability and validity of these subgroups were not yet investigated.

To increase the likelihood of finding clinically relevant and valid autism subgroups, we took several measures that also distinguish the current study from earlier subgrouping attempts. First, we include multiple self-report measures of autism characteristics and of demographic, psychological, and lifestyle variables as input for our analyses. The variables chosen as input are important for the expected utility of the obtained subgroups. We chose measures based on research literature and discussion with autistic adults, that can be easily administered on a large scale. Also, many of these variables are modifiable in nature. Consequently, we increase the likelihood that our findings will be informative for clinical practice. In addition, these variables are selected based on their relevance to the outcomes of interest (i.e., cognitive difficulties, psychological difficulties, and quality of life (QoL)). Second, for *external* validation of the observed subgroups, we focus on the aforementioned outcomes as these are important to general aging (Beydoun et al., 2014; Chen et al., 2014; Goh et al., 2012; Prenderville et al., 2015). Moreover, these variables are clinically relevant and meaningful to autistic adults as well (Howlin & Magiati, 2017). Third, we include two separate samples which together include over 800 adults aged 30–89 years to ensure we can perform a *direct replication* of our results. Fourth, we include both autistic adults and non-autistic adults without ADHD to see whether the observed heterogeneity in autism is distinct from variation we see in non-autistic adults (i.e., whether the observed variation is better described by diagnostic categories than by a continuum across groups). Fifth, we investigate the *specificity* of the results to autism by repeating analyses with inclusion of a group of adults with an ADHD diagnosis; a neurodevelopmental condition which has shown strong overlap with autism in characteristics, as is also acknowledged in the DSM-5 (Antshel & Russo, 2019; Lau-Zhu et al., 2019). Thus, we aim to determine whether valid, replicable, and specific autism subgroups can be identified that are informative for potential future support needs as one ages**.**

## Methods

### Participants

In total, 924 adults participated in this study. Before applying exclusion criteria, the autism group consisted of 509 adults, the comparison (COMP) group of 486 adults and the ADHD group of 124 adults. Please note that we use the term comparison group to indicate non-autistic adults without ADHD.

For all groups, we applied the following exclusion criteria: (a) intellectual disability, (b) insufficient understanding of Dutch language required to complete the questionnaires, (c) age lower than 30 years. In the autism group, we only included participants with a clinical DSM-III, DSM-IV of DSM-5 diagnosis of an Autism Spectrum Disorder (American Psychiatric Association, 1987, 2000, 2013). Most of the included autistic adults received their diagnosis relatively late in adulthood (see Table 1 for details). For the COMP group, we applied additional exclusion criteria to increase the likelihood of a representative non-autistic comparison group without ADHD: (a) a history of more than one psychotic episode, (b) present or past diagnosis of ADHD or a score of six or higher on the Dutch version of the ADHD DSM-IV Rating Scale (Kooij et al., 2005), (c) present or past diagnosis of ASD or total score higher than 32 on the Autism Spectrum Quotient (Baron-Cohen et al., 2001), (d) diagnosis of ASD in close family members (i.e., parent(s), child(ren), brother(s), sister(s)), (e) ADHD diagnosis in close family members. In the ADHD group, we only included adults with a clinical DSM-IV or DSM-5 diagnosis of ADHD and without a clinical ASD diagnosis. All exclusion criteria were checked based on data from self-report questionnaires. Based on the criteria 980 participants could be included (410 autism, 446 COMP, 124 ADHD). The most prevalent reason for exclusion was a score higher than the cutoff on the ADHD Rating Scale, which only applied to adults in the COMP group (i.e., 40 out of 40 exclusion cases). In total, 843 participant had sufficient data to be included in this study (375 autism, 345 COMP, 123 ADHD).

**Table 1:** Sample characteristics

	Autism	COMP	ADHD	Total
*Original data*				
N	114	58	–	172
Sex (M/F)	72/42	33/25	–	105/67
Mean (SD; range)				
Age	54.2 (12.1; 31–89)	56.0 (10.7; 34–79)	–	54.8 (11.6; 31–89)
AQ total	34.6 (6.8; 14–47)	12.6 (4.8; 3–24)	–	27.1 (12.1; 3–47)
ADHD Att sum	10.1 (5.4; 1–25)	4.2 (3.7; 0–17)	–	8.1 (5.6; 0–25)
ADHD Hyp-Imp sum	10.6 (6.1; 1–29)	4.3 (3.7; 0–16)	–	8.5 (6.2; 0–29)
Age of autism diagnosis	48.0 (12.5;12–81)	–	–	–
*Replication data*				
N	261	287	123^b^	671
Sex (M/F/Other)	127/133/1	157/130/0	74/49/0	358/312/1
Mean (SD; range)				
Age	51.2 (12.7; 30–84)	55.7 (13.9; 30–85)	51.2 (11.5; 30–80)	53.1 (13.2; 30–85)
AQ total	34.9 (7.7; 10–48)	13.6 (5.9; 2–31)	20.6 (6.8; 6–40)	23.1 (11.8; 2–48)
ADHD Att sum	12.0 (6.5; 0–30)	5.4 (3.5; 0–17)	18.7 (5.8; 2–31)	10.4 (7.2; 0–331)
ADHD Hyp-Imp sum	12.7 (6.3; 0–32)	6.4 (3.7; 0–23)	19.0 (6.6; 4–31)	11.2 (7.2; 0–32)
ADOS-2 total^a^	11.61 (3.6; 4–19)	–	–	–
Age of autism diagnosis	44.9 (13.4; 4–79)	–	–	–

^a^Sample size is lower for this variable (*N* = 97), since only a subset of participants was administered the ADOS-2

^b^We only included adults with ADHD in the replication data set, because the ADHD data was collected congruently with the replication data

M = male, F = female, AQ = total Autism-Spectrum Quotient total score, ADH = Att sum ADHD Rating Scale, Attention sum score, ADHD Hyp-Imp sum = ADHD Rating Scale, Hyperactivity Impulsivity sum score, ADOS-2 total = Autism Diagnostic Observation Schedule, Module 4 total score

We divided our data into two subsets: an original data set of 172 adults and a replication data set of 671 adults (see Table 1 for sample characteristics). The original and replication data sets, each with different participants, were collected during two different waves (i.e., Wave 2 and 3) as part of a larger longitudinal study on aging and autism (Geurts et al., 2021). The original data set (i.e., Cohort 2) was collected during Wave 2, from December 2015 to December 2016. The replication data set (i.e., Cohort 3) was collected during Wave 3, from September 2018 until October 2020.

Part of the sample (346 in total; 165 autism, 148 COMP, 87 ADHD) was tested with two subtests (i.e., Vocabulary and Matrix Reasoning) of the Wechsler Adult Intelligence Scale-IV (WAIS-IV; Wechsler, 2012). For participants in the autism group, we administered the Autism Diagnostic Observation Schedule—Second Edition (ADOS-2) Module 4 (Lord et al., 2000, 2012).

Autistic and ADHD participants were recruited through mental health institutions in the Netherlands and advertisements placed on client organization websites and social media. COMP participants were recruited via advertisements on social media and within the social environment of the researchers and research assistants of this study. We also consulted our think tank of older/autistic adults for recruitment strategies.

### Measures

We included cluster variables considering whether (a) the variable is potentially predictive of cognitive status and/or comorbid psychological difficulties and/or quality of life in autistic adults, (b) there are known individual differences in scores among autistic adults, (c) the variable is easy to measure on a large scale, so it could be implemented in clinical practice, and whether (d) the variable is either directly or indirectly modifiable to ensure clinical applicability. This resulted in 14 cluster variables that were easy to measure and for which at least two of the other aforementioned questions were answered affirmatively. All measures had sufficient psychometric qualities based on the general population, as described below. We tested the psychometric properties for our autism sample, which resulted in acceptable to good internal consistency for most measures (see Online Resource 1).

#### Cluster Variables

*Autism characteristics* were measured by the Autism Spectrum Quotient (AQ; Baron-Cohen et al., 2001; Hoekstra et al., 2008) as AQ scores have previously been related to QoL (Pisula et al., 2015) and scores are diverse among autistic adults. The AQ consists of 50 items rated on a 4-point scale from “definitely agree” to “definitely disagree”. The items divide into five subscales with 10 items each. We included subscale scores for Social Skills, Attention Switching, Attention to Detail, Communication and Imagination which can vary between 0 and 10 (Baron-Cohen et al., 2001). The internal consistency of the AQ is acceptable with Cronbach’s alpha ranging between 0.63 and 0.77 for subscale scores (Baron-Cohen et al., 2001; Hoekstra et al., 2008).

*Educational level* was measured by asking participants about the highest educational degree they obtained, as lower educational attainment is related to memory problems when aging (Beydoun et al., 2014), and there are differences in educational level among autistic adults (Frank et al., 2018). We used the Dutch Verhage scale to classify the educational level (Verhage, 1964). This scale consists of seven categories that range between 1 (i.e., less than 6 years of primary education) and 7 (i.e., university degree).

*Mastery*—the extent to which we see ourselves as being in control of factors that affect our lives—was assessed with the Pearlin Mastery Scale (Pearlin et al., 1981). Mastery plays a central role in connecting autism traits and depressive symptoms (van Heijst et al., 2020), and autistic adults experience different levels of mastery (Nguyen et al., 2020). The scale consists of seven items rated on a five-point scale from “strongly disagree” to “strongly agree”. We calculated a sum score ranging between 7 (low sense of mastery) and 35 (high sense of mastery). This instrument has a reasonable to high reliability with Cronbach’s α between 0.67 and 0.80 (Penninx et al., 1997; Peterson, 1999).

*Worries/fears:* We used a combination of the Worry Scale (Wisocki et al., 1986) and Fear Questionnaire (Marks & Mathews, 1979). Autism and depression are connected through worry symptoms (van Heijst et al., 2020), and autistic adults experience different levels of worries. This questionnaire includes 15 items that are rated on a five-point scale from “never worries me” to “worries me much of the time”. We calculated a total score ranging between 15 (low worries/fears) and 75 (high worries/fears). This instrument has a good internal consistency and test–retest reliability (Van Der Veen et al., 2014).

*Physical activity***:** We used the International Physical Activity Questionnaire (IPAQ) to measure the amount of physical activity (Craig et al., 2003). Physical activity is an important predictor of QoL in autistic adults and differences exist in the physical activity level of autistic adults (Conn et al., 2011; Hamm & Yun, 2019). The IPAQ includes items about the total time spent in four physical activity domains (i.e., occupational, transport, household and leisure-related physical activity). Physical activities included walking, moderate and vigorous activities for at least 10 consecutive minutes. We calculated the total amount of time (in minutes) during which a participant was physically active during the past seven days. The IPAQ has a good test–retest reliability (Spearman correlation coefficients around 0.80; Craig et al., 2003).

*Negative life events***:** We used the List of Threatening Experiences to measure the number of negative life events in the past year (Brugha et al., 1985), as this number varies between autistic adults and they form a risk for psychological difficulties and lower QoL (Bishop-Fitzpatrick et al., 2017; Rumball et al., 2020). Participants were asked to report whether they experienced any of 12 different life events (e.g., death of a close family member or becoming unemployed). We calculated a sum score ranging between 0 (no threatening life events experienced) to 12 (many threatening life events). The questionnaire has a high test–retest reliability (Brugha & Cragg, 1990).

*Emotional support***:** The Close Persons Questionnaire (CPQ) measures the amount of emotional support received (Stansfeld & Marmot, 1992), which is a predictor of QoL (Khanna et al., 2014; Mason et al., 2018) and levels of emotional support differ among autistic adults (Alvarez-Fernandez et al., 2017). The CPQ includes questions about one’s social network and quality of support representing different categories of support (i.e., informational, emotional, practical, and appraisal). Items are rated on a five-point scale from “never” to “very often”. We calculated a sum score based on 12 items related to emotional support, ranging between 12 and 60. Higher scores indicate higher levels of received emotional support. The four subscales show moderate to good reliability (Hanssen et al., 2019).

*Sensory sensitivity*: The Sensory Sensitivity Questionnaire (SSQ) measures the amount of sensory sensitivity (Lever & Geurts, 2013; Minshew & Hobson, 2008), as sensory sensitivities are related to anxiety levels (Syu & Lin, 2018) and autistic adults report different levels of sensory sensitivity (Kuiper et al., 2019). For each of the 13 items, participants indicated whether they experienced the specific sensory sensitivity (i.e., yes or no). We calculated a sum score between 0 and 13. Higher scores indicate a higher sensory sensitivity level. The Dutch version of the SSQ has an acceptable to good reliability, Cronbach’s α = 0.77 (Lever & Geurts, 2013).

*Positive and negative affect***:** We administered the Positive and Negative Affect Schedule (PANAS; Watson et al., 1988), as negative affect and affective instability have been linked to depression in autistic adults, and scores on affect are diverse among autistic adults (Dallman et al., 2021). Positive Affect (PA) represents the extent to which we feel enthusiastic, alert, and active. Negative Affect (NA) represents subjective distress encompassing a variety of aversive mood states (e.g., fear, anger, disgust). The scale consists of 20 feelings or emotions (i.e., 10 measuring PA and 10 measuring NA) that are rated on a five-point scale from “very slightly or not at all” to “extremely”. We calculated subscale scores for PA and NA ranging between 10 and 50. The subscales have a high reliability (Watson et al., 1988).

#### Variables for External Validation

We compared the obtained subgroups on cognitive difficulties, psychological difficulties and QoL.

*Cognitive difficulties*: The Cognitive Failures Questionnaire (Broadbent et al., 1982) is a valid and reliable questionnaire to measure the amount of cognitive difficulties (vom Hofe et al., 1998). This questionnaire consists of 25 items rated on a five-point scale from “never” to “very often”. The total score ranges between 0 and 100; higher scores indicate more cognitive difficulties. “An example of an item is: “Do you read something and find you haven’t been thinking about it and must read it again?”. A different example is: “Do you forget whether you’ve turned off a light or a fire or locked the door?”. The questionnaire has a good test–retest reliability (Bridger et al., 2013).

*Psychological difficulties:* We used the Symptom Checklist-90-Revised (SCL-90-R) to measure psychological difficulties (Derogatis, 1977). The SCL-90-R consists of 90 items that are rated on a five-point scale. We calculated the total score and scores on nine subscales (i.e., agoraphobia, anxiety, depression, somatization, cognitive performance deficits, interpersonal sensitivity, hostility, sleep difficulties and items not included in any specific factor). A higher score is indicative of more psychological difficulties. The Dutch version of the SCL-90-R has a high reliability (Smits et al., 2015).

*Quality of life:* The World Health Organization Quality of Life Questionnaire-BREF (WHOQoL-BREF) was used to measure QoL (THE WHOQOL GROUP, 1998). This questionnaire has 26 items rated on a five-point scale indicating how someone has felt during the past two weeks. We calculated scores on four subscales (i.e., physical health, psychological, social relationships and environment). Higher scores indicate a higher quality of life. The instrument has good psychometric properties (McConachie et al., 2018).

### Procedure

For the precise procedure we refer to the published protocol (Geurts et al., 2021). In short, interested participants were contacted via telephone, e-mail, or written letters. After obtaining written informed consent participants first filled out questionnaires either online or on paper depending on the participant’s preference. Participants required around two hours to complete the questionnaires. Second, a subset of participants was interviewed either online or in person (including questions regarding psychotropic medication use and depending on one’s diagnostic category, the ADOS-2) and tested (e.g., shortened WAIS-IV). Neuropsychological testing was also part of the procedure for a subset of participants, but those data were not included here. Participants received €7,50 for filling out the questionnaires and €10,00 for the interview/test session. They also received a maximum of €20,00 for their travel expenses. This study was approved by the local ethical review board of the department of Psychology of the University of Amsterdam (Wave 2: 2015-BC-4270 and Wave 3: 2018-BC-9285).

### Statistical Analyses

All analyses were conducted in RStudio version 1.3.1073 (RStudio Team, 2020), using the R-package *igraph* for subgroup identification (Csardi & Nepusz, 2006).

The analysis plans for the original and replication data were preregistered at AsPredicted.org (AsPredicted #29596 (https://aspredicted.org/xx6u8.pdf) and #34234 (https://aspredicted.org/e7f86.pdf)).

#### Missing Data

Within a questionnaire specific items can be missing. At the item level, we considered 10% of missing data per participant appropriate for imputation (Bennett, 2001). The type of imputation depended on the specific measurement instrument. For mastery, autistism characteristics, sensory sensitivity, worries/fears, positive and negative affect, and emotional support, we recoded a maximum of 10% of missing values to the median of the participant’s other responses on this specific questionnaire. For negative life events and physical activity, we recoded a maximum of 10% of missing values to zero, implying the absence of a negative life event or the absence of a specific physical activity. We did not impute missing values on education.

A full questionnaire might also be missing. At the instrument level, participants with no more than one missing value were included and such missing values were not imputed. Hence, for each included individual we had information on at least 13 cluster variables.

#### Community Detection Analysis

For the community detection analyses, we first transformed scores on all cluster variables to *z*-scores, such that differences in measurement scales of the instruments would not influence the subgrouping results. Please note that this did not involve a normalizing z-transformation, hence the shape of the distribution of scores was not impacted by this transformation (e.g., Box & Cox, 1964; Milligan & Cooper, 1988). We then created a pairwise Pearson correlation matrix, including person-to-person relationships between all pairs of participants in the study sample. A high correlation in this matrix indicates that two participants have similar scoring patterns on the cluster variables (Karalunas et al., 2014).

In the resulting network each node represents a participant and the edges connecting the nodes represent the correlations between scoring patterns of pairs of adults. We aimed to identify communities (or subgroups), which are locally dense connected subgraphs in the larger network (Barabási & Pósfai, 2016). Participants (i.e., nodes) belonging to a community have a higher probability of connecting to other members of that community than to participants of a different community. Different algorithms can be used for a community detection analysis. The Spinglass algorithm (Reichardt & Bornholdt, 2006) was preferred over others (Radhoe et al., 2021), because, amongst other things, this algorithm is able to deal with weighted egdes (in this case, correlations). Moreover, it takes both positive and negative correlations into account, which is important since we aimed to avoid inclusion of dissimilar participants (i.e., with opposite scoring patterns) in the same community. Also, with the Spinglass algorithm each participant is assigned to a single community. This ensures that the resulting communities could eventually become informative for clinical practice, where autistic adults could be aided to transfer from one community to another (possibly more favorable) community. The gamma-parameter was set to 1.0 to assign equal importance to present and non-present edges between adults.

In addition to the preregistered analyses, the modularity index *Q* was calculated quantifying the quality of the assignment of participants into communities (Newman & Girvan, 2004). The *Q*-index indicates the differences between (a) the true connections in a network, and (b) the connections that would be expected if the network was randomly wired (Barabási & Pósfai, 2016). Positive *Q*-values suggest that there are more connections than would be expected by chance, representing a potential community structure. A *Q*-value of 0 implies that the connections between nodes are completely random, and negative *Q*-values suggest that the nodes do not form a community. Higher *Q-*values indicate a stronger community structure. In practice, *Q* ranges from 0.3 to 0.7, with a maximum value of 1.

#### Direct Replication and Specificity

We performed the community detection analysis in different steps. First, we performed the community detection analysis using the original data set (i.e., autism and COMP). Second, a direct replication of our community detection analysis was conducted using the replication data set containing an independent group of autistic and COMP participants. As subgrouping techniques—including community detection—are potentially susceptible to over-fitting and generalization issues, it is important to validate the results using a larger replication sample (Bubeck & von Luxburg, 2007; Horne et al., 2020). The goal was to determine whether the same subgroups could be identified in a second sample, which would support the subgroups’ validity. Third, the analyses on the replication data set were repeated while also including participants with ADHD to test the specificity of the observed findings. The subgrouping solution was considered specific to autism if not all participants with ADHD were allocated to the same subgroup as autistic participants, i.e., ADHD participants would form a separate subgroup or participants with ADHD would be allocated to the COMP subgroup.

#### External Validation

Subgroups were compared on variables not included in the community detection analyses to determine the validity of the subgrouping results. We considered the results meaningful if the identified subgroups differed significantly on the external variables.

First, an ANOVA or t-test (depending on the number of identified subgroups) was used to assess whether the subgroups differ in their experience of cognitive difficulties. Second, we used ten ANOVA’s or t-tests to assess whether the subgroups differ in reported psychological difficulties (i.e., SCL-90-R total score, and nine subscale scores). Third, differences in QoL between the identified subgroups were assessed with four ANOVA’s or t-tests. In addition to our preregistered analyses, we performed two MANOVA’s with subscale scores as dependent variables for QoL and psychological difficulties. To correct for multiple testing, we divided our threshold for significance by ten as this (a) corresponded to the maximum number of comparisons within one domain (i.e., ten tests were used for the psychological difficulties domain), and (b) would correspond to “substantial” or “strong” evidence in terms of Bayesian classifications (Benjamin et al., 2018). Thus, *p* < 0.005 was used as the threshold for statistical significance.

### Community Involvement

For this study, and our overall study on aging in autism (Geurts et al., 2021), we worked together with a group of four older/autistic adults, also referred to as the “think tank”. We met at least three times a year (either online or in person) to discuss, among other things, recruitement strategies, information letters and the interpretation of study results. For this specific study, the think tank also made suggestions for the interpretations of the subgroup findings and decided the naming of the obtained subgroups during two online meetings. The members were paid for their contribution.

## Results

After checking the exclusion criteria and dealing with missing data, the original data set included 172 adults (114 autism, 58 COMP). The replication data set included 671 adults (261 autism, 287 COMP, 123 ADHD). Although there is no formal way of establishing the required sample size for community detection yet, the present sample size seems sufficiently large given (a) simulations described in Geurts et al. (2021) and Agelink van Rentergem et al. (2022), and (b) previous studies adopting a community detection approach including similar sample sizes (Blanken et al., 2020; Karalunas et al., 2014; Mostert et al., 2018). The amount of missing data (a) in total, and (b) per cluster variable is described in Online Resource 2. A correlation matrix of the cluster variables can be found in Online Resource 3. The distribution of scores on the cluster variables for the autism and COMP groups based on the replication data are provided in Online Resource 4.

### Autistic and Non-autistic Adults form Separate Subgroups

We identified two subgroups (*Q* = 0.41). The subgroups correspond to autism and COMP as one subgroup (*N* = 81) mainly included COMP participants (i.e., 70%), whereas the other (*N* = 91) mainly included autistic adults (i.e., 99%). Subgroup profiles on the cluster variables are depicted in sFigure 2 in Online Resource 5. In line with our preregistration, we followed this result up with a separate community detection analysis for the autism group to gain more insight into the heterogeneity within autism.

### Autistic Adults form Three Separate Subgroups

**Fig. 1:**
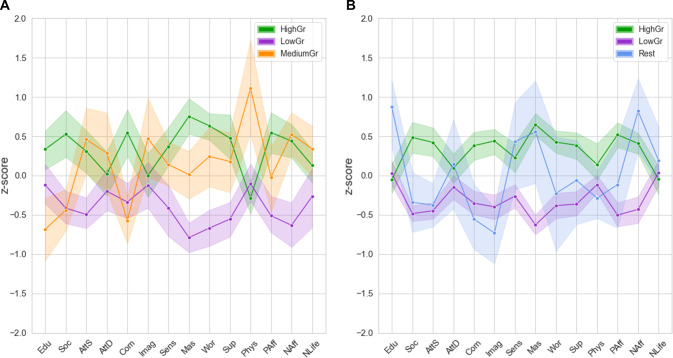
**A** Three autism subgroup profiles based on original data. **B** Three autism subgroup profiles based on replication data. *Note*. HighGr = Feelings of high grip, LowGr = Feelings of low grip, MediumGr = Feelings of medium grip with high physical activity, COMP = comparison, Edu = education, Soc = social skills, AttS = attention switching, AttD = attention to detail, Com = communication, Imag = imagination, Sens = sensory sensitivity, Mas = mastery, Wor = worry, Sup = emotional support, Phys = physical activity, PAff = positive affect, NAff = negative affect, NLife = negative life events. Higher z-scores represent higher scores on Edu, Soc, AttD, AttS, Com, Imag, Mas, Sup, Phys, PAff. Higher z-scores represent better scores on Sens, Wor, NAff, NLife (less sensitivity, less worrying, less negative affect, fewer negative life events). Shaded area represents 95%-confidence interval. Profile of comparison group is plotted as a reference (dotted line). Since we did not replicate the MediumGr subgroup in the replication data, we labelled the remaining subgroup of *N* = 7 adults as “Rest” in panel B

Three distinct autism subgroups were identified (*Q* = 0.30). Figure 1 Panel A depicts subgroup profiles on the cluster variables in the original data. After consulting with our older/autistic think tank, the labels of the subgroups were based on the cluster variables on which the subgroups differed significantly. The first subgroup (*N*_1_ = 49, 43%) was characterized by the highest educational level, highest scores on social skills and communication (i.e., low scores on AQ subscales), highest sense of mastery (i.e., feeling of being in control and having a grip on what is happening in your life) and highest level of positive affect. Our think tank suggested the term “*Feelings of high grip*” (HighGr) for this subgroup, to indicate that people in this subgroup experience more control over what happens in their life, which also corresponds to the higher social skills, positive affect and lower worries these autistic adults report.

The second subgroup (*N*_2_ = 48, 42%) differed from the other subgroups on social domains, mastery, worry, support and affect. This subgroup was characterized by the lowest scores on attention switching, lowest sense of mastery, and highest levels of worries and negative affect. The term “*Feelings of low grip*” (LowGr) was suggested by the think tank, as adults in this subgroup reported less control over what happens in their life, reflected by lower scores on mastery, the social domain, positive affect, and higher scores on worries and negative affect. We labeled the third subgroup (*N*_3_ = 17, 15%) as the “ *Feelings of medium grip with high physical activity*” (MediumGr) subgroup. Participants in this subgroup were characterized by the lowest level of education, low scores on communication and social skills, average level of mastery, and the highest level of physical activity.

### Direct Replication of Separate Subgroups Autistic and Non-autistic Adults

**Fig. 2:**
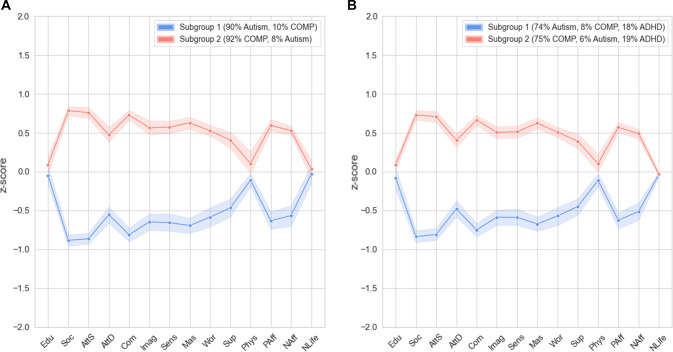
**A** Subgroup profiles based on data from the autism and COMP groups for each of the two community detection-based subgroups formed on replication data. **B** Subgroup profiles based on data from the autism, COMP, and ADHD groups for each of the two community detection-based subgroups formed on replication data. *Note*. COMP = comparison, Edu = education, Soc = social skills, AttS = attention switching, AttD = attention to detail, Com = communication, Imag = imagination, Sens = sensory sensitivity, Mas = mastery, Wor = worry, Sup = emotional support, Phys = physical activity, PAff = positive affect, NAff = negative affect, NLife = negative life events. Higher z-scores represent higher scores on Edu, Soc, AttD, AttS, Com, Imag, Mas, Sup, Phys, PAff. Higher z-scores represent better scores on Sens, Wor, NAff, NLife (less sensitivity, less worrying, less negative affect, fewer negative life events). Shaded area represents 95%-confidence interval

Combining the data of participants with and without autism again resulted in two larger subgroups (and a third subgroup consisting of one person) (*Q* = 0.43, which is comparable to the *Q*-value found in the original data set). After excluding the third subgroup consisting of one person, the *Q*-value remained similar, i.e. *Q* = 0.43. Moreover, we replicated the finding that the two remaining subgroups mainly indicated a distinction between autism and COMP as one subgroup (*N* = 265) mostly included autistic adults (90%), whereas the other subgroup (*N* = 282) mostly included COMP participants (92%) (Fig. 2, Panel A). As preregistered, we again performed a separate community detection analyses for the autism group to gain insight into the heterogeneity within this group.

### Direct Replication of Two Out of Three Subgroups of Autistic Adults

We replicated our findings by identifying three distinct autism subgroups (*Q* = 0.28, which is similar to the *Q*-value found in the original data set). However, subgroup profiles of only two out of three subgroups were similar to those obtained in the original data. Subgroup profiles are depicted in Fig. 1 Panel B. We again identified a “*Feelings of high grip*” subgroup (*N*_1_ = 124, 47%) that was characterized by the highest scores on social skills, attention switching, communication, and positive affect. The “*Feelings of low grip*” subgroup (*N*_2_ = 130, 50%) was also replicated, characterized by the lowest sense of mastery and highest level of negative affect. The third subgroup (*N*_3_ = 7, 3%) was characterized by the highest educational level. The profile of this subgroup did not resemble the third profile identified in the original data set; i.e., we did not replicate the “*Feelings of medium grip with high physical activity”* subgroup. Also, only seven autistic adults were included in this subgroup. Therefore, we did not consider this a separate subgroup and did not include this subgroup in further analyses in the manuscript. Nonetheless, as all group comparisons were preregistered, results (including descriptive statistics) regarding this Rest-subgroup are included in Online Resource 6, but should not be used to draw conclusions about the Rest-subgroup (Stevens, 1996). In addition, we recalculated the modularity index by exluding participants in the third subgroup, which hardly changed the strength of the community structure (i.e., *Q*_old_ = 0.28 and *Q*_new_ = 0.29).

Table 2 presents test statistics for differences between the two main autism subgroups. Because test statistics are describing the group differences rather than testing hypotheses, we did not correct these values for multiple testing. The subgroups differed on 11 out of 14 cluster variables, but not on level of education. The subgroups did not differ in age, sex, or IQ, suggesting that subgroup differences were not driven by demographic or IQ differences.

**Table 2:** Raw cluster variable scores and descriptives for the two major autism subgroups formed on replication data (*N* = 254)

	Subgroup			
	HighGr	LowGr		
	*N* = 124	*N* = 130		
Variable	*M (SD);* range	*M (SD);* range	Test Statistic	Effect size (*η*^*2*^)
*Cluster variables*				
Education^a^	5.9 (0.9); 2–7	6.0 (0.8); 3–7	*F*(1, 249) = 0.4	< 0.01
AQ social skill	6.4 (2.5); 0–10	8.6 (1.4); 4–10	*F*(1, 252) = 77.6***	0.24
AQ attention switching	6.9 (2.2); 0–10	8.7 (1.3); 4–10	*F*(1, 252) = 61.7***	0.20
AQ attention to detail	6.4 (2.4); 0–10	7.0 (2.0); 2–10	*F*(1, 252) = 3.9	0.02
AQ communication	5.9 (2.3); 1–10	7.6 (1.9); 0–10	*F*(1, 252) = 38.4***	0.13
AQ imagination	5.0 (2.0); 1–10	6.8 (2.0); 1–10	*F*(1, 252) = 55.0***	0.18
Sensory sensitivity	6.5 (2.8); 0–13	7.8 (2.3); 1–13	*F*(1, 247) = 15.6***	0.06
Mastery	23.2 (4.3); 14–35	16.5 (3.8); 7–25	*F*(1, 251) = 172.6***	0.41
Worry	29.0 (9.6); 15–64	38.4 (11.3); 20–69	*F*(1, 252) = 50.5***	0.17
Emotional support	33.1 (10.7); 12–53	24.8 (10.1); 12–54	*F*(1, 245) = 40.0***	0.14
Physical activity^b^	1314.8 (2028.1); 0–18,050	908.8 (1022.0); 0–5010	*F*(1, 247) = 4.0*	0.02
Positive affect	32.5 (6.4); 19–49	25.0 (6.2); 10–42	*F*(1, 252) = 88.5***	0.26
Negative affect	18.4 (6.7); 10–40	25.5 (8.4); 10–45	*F*(1, 252) = 54.4***	0.18
Negative life events	0.8 (1.1); 0–5	0.7 (1.0); 0–5	*F*(1, 249) = 0.5	< 0.01
*Descriptive variables*				
Age	50.6 (13.8), 30–81	51.3 (11.6), 30–84	*F*(1, 252) = 0.21	< 0.01
*Biological sex*^c^			χ^2^(2) = 3.0	
% male	53	44		
% female	47	55		
IQ score^d^	116.7 (17.2)	113.4 (15.3)	*F*(1, 92) = 1.0	0.01

AQ = Autism-Spectrum Quotient, HighGr = Feelings of high grip, LowGr = Feelings of low grip

*p < 0.05, **p < 0.01, ***p < 0.001

^a^Dutch Verhage scale was used to classify the educational level

^b^Physical activity is measured in minutes

^c^The remaining percentage was classified as “other”

^d^Sample size (*N* = 165) is lower for this variable because data are only available for participants who completed the interview. Please note that we did not ask participants about race/ethnicity and socioeconomic status, but the majority of the participants was White and had a high educational attainment

### Two Replicated Autism Subgroups Differ on All External Validators

External validation results for the original data are presented in Online Resource 7, sTable 5. The LowGr subgroup indicated significantly more cognitive difficulties and more psychological difficulties when compared to the HighGr subgroup. The LowGr subgroup also scored significantly lower on all measures of QoL. In addition to the univariate test results, multivariate tests indicated significant differences in psychological difficulties and quality of life between the subgroups.

### Replication of External Validation: LowGr Subgroup Scores Less Favorably on all External Validators

External validation results are presented in Table 3 (and results including the smaller third autism subgroup are presented in Online Resource 8, sTable 6), and are similar to the results obtained from the original data set. The LowGr subgroup again reported significantly more cognitive difficulties than the HighGr subgroup. Moreover, the LowGr subgroup scored significantly higher on all measures of psychological difficulties, and significantly lower on all measures of QoL. This was also indicated by multivariate tests, as there was a significant difference in psychological difficulties and quality of life between the two subgroups.

**Table 3:** External validation measures for the two major autism subgroups based on replication data (*N* = 254)

	Subgroup			
	HighGr	LowGr		
	*N* = 124 (47%)	*N* = 130 (50%)		
Variable	*M*(*SD*); range	*M*(*SD*); range	Test statistic	Effect size (*η*^*2*^)
Cognitive difficulties	43.1 (14.8); 12–90	51. 8 (13.8); 17–88	*F*(1, 252) = 19.5*	0.07
SCL-90 total score	149.4 (41.0); 93–337	199.3 (52.5); 95–397	*F*(1, 249) = 70.3*	0.22
SCL-90 anxiety	15.9 (5.8); 10–43	21.6 (7.7); 10–48	*F*(1, 251) = 42.9*	0.15
SCL-90 agoraphobia	9.3 (3.1); 7–24	12.9 (5.1); 7–34	*F*(1, 251) = 45.1*	0.15
SCL-90 depression	28.0 (9.1); 16–61	40.6 (13.1); 16–76	*F*(1, 251) = 78.3*	0.24
SCL-90 somatization	19.2 (6.6); 12–47	22.5 (7.7); 12–51	*F*(1, 249) = 13.3*	0.05
SCL-90 cognitive performance deficits	18.0 (6.2); 9–41	23.6 (6.8); 9–41	*F*(1, 251) = 45.8*	0.15
SCL-90 interpersonal sensitivity	30.0 (10.0); 18–80	41.1 (13.0); 19–79	*F*(1, 250) = 58.2*	0.19
SCL-90 hostility	8.7 (3.3); 6–29	10.4 (4.0); 6–28	*F*(1, 251) = 13.5*	0.05
SCL-90 sleep difficulties	6.6 (2.9); 3–14	8.5 (3.5); 3–15	*F*(1, 251) = 22.3*	0.08
SCL-90 rest	13.8 (4.7); 9–40	17.7 (5.5); 9–34	*F*(1, 251) = 37.2*	0.13
QoL Physical health	14.3 (2.6); 7–19	12.1 (2.6); 6–20	*F*(1, 250) = 45.0*	0.15
QoL Psychological	13.4 (2.3); 9–20	10.8 (2.4); 5–19	*F*(1, 251) = 81.0*	0.24
QoL Social relationships	12.9 (2.8); 7–20	10.7 (3.2); 4–19	*F*(1, 252) = 34.4*	0.12
QoL Environment	15.8 (2.1); 11–20	14.1 (2.4); 8–19	*F*(1, 251) = 38.9*	0.13
*Multivariate analyses*^a^				
SCL-90			*F*(9, 241) = 10.8^b^	
QoL			*F*(4, 246) = 22.8^b^	

HighGr = Feelings of high grip, LowGr = Feelings of low grip, SCL-90 = symptom checklist, QoL = World Health Organization Quality of Life Questionnaire-BREF

^*^p < 0.005

^a^Wilks’ Lambda

^b^*p* < 0.05

### Testing Specificity: Addition of ADHD Group Does Not Alter Subgrouping Solution

After adding a group of adults with an ADHD diagnosis to investigate specificity, we again detected two subgroups (*Q* = 0.42). Once more a distinction was found between the autism group and COMP group, as the first subgroup (*N*_1_ = 321) mostly included autistic adults (239/321, 74%) and the second subgroup (*N*_2_ = 346) mostly included non-autistic adults (261/346, 75%). The ADHD group was almost equally distributed among these two subgroups, as 57 (47%) adults with ADHD belonged to the first subgroup and 64 (53%) adults with ADHD belonged to the second subgroup. Inspection of subgroup profiles (Fig. 2, Panel B) indicated that the two identified subgroups are identical to the two identified subgroups when adults with ADHD were not included (Fig. 2, Panel A). Thus, addition of an ADHD group did not alter the earlier observed subgrouping solution.

## Discussion

In this study, we identified subgroups in adults with autism or ADHD, and comparison participants using self-report measures of autism characteristics, and demographic, psychological, and lifestyle variables. Community detection analysis based on data from comparison participants and autistic adults indicated two distinct subgroups: one of comparison adults and one of autistic adults. We replicated these subgroups in a second data set. This indicates that the variation in variables as diverse as worrying, emotional support and mastery, is better described by diagnostic category than by continuous variation across people (Abu-Akel et al., 2019; Frazier et al., 2010). When we added ADHD adults to this second data set, half of them grouped together with the autism subgroup, and half with the COMP subgroup. Community detection analysis of just autistic adults indicated three subgroups: (a) “*Feelings of high grip*”, (b) *Feelings of low grip*”, and (c) “*Feelings of medium grip with high physical activity”.* Subgroups were particularly distinct in feelings of grip (i.e., mastery), the social domain, and affect. We replicated the profiles of the first two subgroups in our replication data set, and showed that these subgroups differed on the external validators: cognitive difficulties, psychological difficulties, and QoL.

The two autism subgroups we identified and replicated were distinct on most cluster variables. Therefore, it seems that a variety of factors—i.e., not only variables related to self-reported autism characteristics—cause the distinction between subgroups. The “*Feelings of low grip”* subgroup was characterized by a more vulnerable profile on the cluster variables; low scores on the social domain, lowest sense of mastery (i.e., experienced grip on life), and highest level of negative affect. This was also indicated by the external validation, as this subgroup reported more cognitive difficulties, more psychological difficulties and a lower QoL compared to the *“Feelings of high grip”* subgroup. While the subgroups differed on most cluster variables, they were similar on some variables, including education and negative life events. Thus, while the subgroups did not differ on educational level attained and the amount of experienced negative life events, they showed large differences on the other cluster variables, and external validators. These results suggest that the differences between the subgroups are not driven by the *number* of negative/traumatic experiences, but rather by how these experiences are dealt with in daily life, or by the *type* of experiences.

We replicated two out of three autism subgroups. The third “rest” subgroup that did not replicate only included seven adults in our replication data set. We consider this subgroup an artifact of the Spinglass community detection method, rather than a distinct and valid subgroup. Subgrouping techniques, including community detection, are known for over-fitting or failing to generalize (Bubeck & von Luxburg, 2007; Horne et al., 2020). Consequently, it is worrying how validating one’s results in a separate sample is often not included in subgrouping studies in the autism research field (Agelink van Rentergem et al., 2021). The findings of the current study emphasize the importance of a direct replication: Without this validation procedure, we would not have known that this rest subgroup was not a valid subgroup and we could have overinterpreted the findings in the original dataset.

The results on the specificity to autism were somewhat inconclusive. We had anticipated several possible results: had adults with ADHD been similar to comparison participants or formed their own subgroup, this would have suggested specificity of the autism subgroup; had they been similar to autistic adults, this would have suggested nonspecificity of the autism subgroup. However, the adults with ADHD were divided equally across both subgroups. One possible explanation for our findings may be related to an overarching condition perspective of autism and ADHD (van der Meer et al., 2012): Those with the fewest ADHD characteristics are indistinguishable from comparison participants, and those with the most ADHD characteristics are indistinguishable from autistic adults. However, critical examination of our inclusion criteria is also warranted. Autistic adults were allowed to have a comorbid ADHD diagnosis, and 20% of our autism sample did. The ADHD group was not screened for reporting too many autism characteristics on the AQ. Therefore, some overlap between ADHD and the other two categories may have been rooted in the design.

The finding that the autism and COMP subgroups did not differ on education level was unexpected, since the literature often shows that autistic adults attain a lower education level than non-autistic adults (Anderson et al., 2017; Shattuck et al., 2012). There are several possible explanations for this result: (a) we did not include any participants with a diagnosis of intellectual disability, (b) most of the adults in the autism group received a late ASD diagnosis (i.e., 94% in original data and 97% in replication data was diagnosed after age of 18 years), so the included autistic aduls might not have encountered as many problems during their education as compared to people diagnosed in childhood, or might have been able to compensate for their difficulties (Livingston et al., 2020), and (c) highly educated people are more likely to participate in scientific studies (Reinwand et al., 2015; Viken et al., 2019).

It is important to consider the representativeness of our autism sample when interpreting our findings. First, autistic participants were selected based on diagnosis rather than ADOS or AQ scores. Such scores are snapshots of the full behavioral profile and the scores that we report were not obtained throughout the diagnostic process. Hence, we consider it important for the inclusion criteria to follow the clinical diagnosis given the purpose of the current study. Second, most of the adults in the autism group received a late ASD diagnosis. Our knowledge of autism in adulthood is expanding so we are increasingly able to recognize the presentation of autism characteristics in this age group. Nonetheless, we need to be aware that these results may not be generalizable to autistic adults diagnosed in childhood. As indicated by a recent study, there may be differences between autistic adults diagnosed in adulthood and those diagnosed in childhood, especially in co-occurring psychiatric conditions (Jadav & Bal, 2022). Third, our sample was a blend of adults recruited from the community and from mental health institutions. This recruitment strategy was adopted to ensure an accurate representation of the diverse population of autistic adults. Fourth, we only included autistic adults with average to high intelligence. Inclusion of autistic adults with an intellectual disability (ID) would have likely resulted in two autism subgroups: one subgroup with ID and one subgroup without ID. This implies that our subgroup analysis would merely reflect subgroups across intellectual ability, rather than capturing the heterogeneity across demographic, psychological, and lifestyle factors. Thus, given the goal of the current study, we chose to exclude adults with an ID. Hence, our results are probably not generalizable to autistic adults with an intellectual disability.

Moreover, it should be noted that 12% of participants in the original data set and 6% in the replication data set were excluded due to missing data. Missing data mostly occurred on instruments measuring emotional support, physical activity and negative life events. The questionnaire measuring emotional support (Stansfeld & Marmot, 1992), was administered last in the questionnaire booklets, and may have been skipped more frequently by participants. To measure physical activity, a questionnaire was used that is relatively more demanding to fill out. For different types of physical activity, participants had to indicate how much time (in minutes) they spent on a specific physical activity, which is relatively more challenging than the other questionnaires. Nonetheless, this measure has been validated in previous research (Craig et al., 2003). Missing data on the questionnaire measuring negative life events (Brugha et al., 1985) could reflect the traumatic nature of the items, and therefore, could indicate difficulty participants may have had to complete these questions. Moreover, the questionnaires for physical activity and negative life events both include retrospective questions, that require more time from the participants and may, therefore, have been skipped more often.

This study is unique in its sample (e.g., autistic individuals included, sample size, and age span), included measures, analysis, and validation procedure. First, the sample was large compared to what is typical in the autism subgrouping literature (Agelink van Rentergem et al., 2021). Also, we adopted a wider age range, and included both adults with autism, ADHD, and a comparison group. Second, we designed the study in such a way that we had a multivariate data set, which allowed us to include multiple cluster and external variables across different domains. This is important as the goal of our analysis was to detect differences between subgroups in variables that are meaningful to autistic adults: cognitive and psychological difficulties, and quality of life. This also guided the variable selection procedure. Third, the analysis method, Spinglass community detection, has rarely been used in autism. Fourth, we preregistered most analyses. Fifth, we included several validation strategies to critically evaluate our results and to examine the validity of the subgrouping results (Agelink van Rentergem et al., 2021).

With our external validation procedure, the identified autism subgroups were compared on clinically relevant measures related to the cluster variables, that were not used in the community detection analysis itself: cognitive and psychological difficulties, and QoL. Although these cluster variables and external variables are different constructs, some may wonder whether certain variables used to build and test the subgroups (e.g., negative affect and psychological difficulties) are too closely associated and, therefore, being a methodological concern. However, it was a deliberate choice to include both external variables that are more closely related to the cluster variables (i.e., psychological difficulties), and some that are less closely related (i.e., QoL and cognitive difficulties) as they provide different information on the validity of the subgroups. Differences on external variables that are more closely related to the clustering variables suggest that the subgroup differences were structural, i.e., less overfitting of the random noise in the clustering variables in this particular sample. Including QoL and cognitive difficulties demonstrates that subgroup differences also extend to variables less closely related to the cluster variables, highlighting the generalizability of the subgroups.

It is relevant to point out that the use of subgroup labels (e.g., Feelings of high grip) could (mis)guide the interpretation of findings in subgroup research and could potentially affect conclusions. Therefore, we considered it both important and necessary to consult our think tank of older autistic adults for the subgroup labels and conclusions reported in this study. However, even in this case one should be careful not to use the suggested labels outside the context of this study. As our replication sample showed, the subgroups differed on more cluster variables than mastery, so nuances may get lost when using subgroup labels.

Moreover, the labels were based on the mean differences, between the groups, but the assignment of participants to a specific subgroup was not based on the level of the scores, but on the pattern of scores. By calculating the correlations and using this as input, the level of scores is corrected for. Therefore, participants in the same subgroup have a similar pattern of peaks and troughs, even though one participant may have high scores on specific measures, and the other has one low scores on specific measures. Conversely, participants in different subgroups may overall have the same level of scores, but are assigned to different subgroups because there is a double dissociation in where the peaks and troughs in their pattern of scores are (Crawford et al., 2003). To interpret and describe the subgroups, we did examine whether there were level differences in scores as well, and found that these were present on some domains. But because of the way the participants were assigned based on strengths and difficulties, we should not and cannot state that participants in one subgroup show a deficit. The findings of the current study are in line with a categorical difference in autism, rather than a dimensional difference (Abu-Akel et al., 2019; Frazier et al., 2010). For clinical practice, this entails that to correctly apply these findings, clinicians should not focus on ‘severity’ by using cut off scores, but instead focus on the pattern of strengths and difficulties to determine subgroup membership.

Furthermore, it remains difficult to evaluate one’s subgrouping results, as there is no golden standard on how to determine the robustness of the results. In this study, we adopted several preregistered techniques to confirm the validity of our results, which is more than is usually done in the autism research realm (Agelink van Rentergem et al., 2021). Nonetheless, the modularity index, that was calculated in addition to the preregistered analyses, resulted in *Q*-values around 0.30. Although positive *Q*-values are indicative of a potential community structure, the absolute values were relatively low. Even when we ran a community detection analysis including the autism and comparison group—that are known to differ on many cluster variables—the *Q*-index was relatively low (*Q* = 0.41). Similar values have been reported in different community detection studies analyzing psychological data (Blanken et al., 2020; Karalunas et al., 2014; Radhoe et al., 2021). These low values could be due to the inclusion of people in the community detection analysis (as compared to more distinct entities), suggesting that people are overall more similar than different. This could indicate that the modularity index may not be well-suited for psychological data, although this has to be investigated in methodological research.

To be directly applicable to clinical practice, it is essential to first assess how these subgroups develop over time. The subgroups did not differ in age, but this only provides cross-sectional evidence for a lack of a developmental effect. Longitudinal data is needed to determine whether the identified subgroups are stable over time and can be used to make clinical predictions. Therefore, we collected follow-up data and aim to assess the temporal stability of the subgroups, and their predictive value for future clinical outcomes (Geurts et al., 2021). If the subgroups’ validity proves robust in these additional validation steps, we can turn towards the development of interventions. For example, future studies could investigate whether the relationship among characteristics differs between the two subgroups. If differences are found in relationships between characteristics, future research may address whether intervening on the characteristics that differ most strongly between the subgroups, results in transitions in subgroup membership, or whether interventions should focus on the relationship between characteristics instead. Moreover, in this study we have focused on self-report questionnaire data; future work could also include proxy or clinican report for a more comprehensive picture. Furthermore, qualitative data can also enrich the interpretation of the current subgroup findings.

In the clinical field, it is recognized that there is a group of autistic adults that reports having feelings of low control, or low sense of mastery. In order to support these autistic adults, in the Netherlands, job coaches or life coaches are often hired to help people gain control over their life. This seems to be useful, but the subgroups formed could also inform us about the level of care needed. In the Netherlands, there is a distinction between general mental health care that is easily accessible for everyone, and specialized mental health care that is directed at specific groups such as those autistic adults for whom their care needs can not be met within general mental health care. The subgroups that we identified in this study could therefore indicate the distinction between autistic adults who could benefit from this highly specialized care (i.e., the “Feelings of low grip” subgroup) and those that might already be helped via basic mental health care (i.e., the “Feelings of high grip” subgroup) when this is needed. Thus, it should be noted that not every autistic adult is in need of highly specialized care. It is also more likely that in the group with higher quality of life and less cognitive and psychological difficulties, there are autistic people who do not have any support needs, as not every autistic adult is in need of mental health care. Moreover, the current study implies that for autistic adults in the LowGr subgroup, vulnerabilities in one domain (e.g., mastery) are often accompanied by other difficulties (e.g., worries or negative affect). Therefore, if an autistic person reports difficulties in one domain, it may be helpful to screen for vulnerabilities in additional domains as we know these are associated with more cognitive and psychological difficulties, and a lower QoL. A better grasp on the full representation of the challenges someone might experience, may be crucial for tailored support to eventually improve the lives of autistic people.

In conclusion, we not only discovered that autistic adults form a clearly distinct group from adults without an autism diagnosis, but also found subgroups among autistic adults when focusing on autism characteristics and demographic, psychological, and lifestyle factors. While we replicated these findings and showed that these subgroups differ on clinically relevant outcomes (i.e., they are externally valid), these subgroups warrant further research to determine the longitudinal stability. Moreover, with this study we show which largely modifiable variables may distinguish these subgroups, which might be a starting point for an intervention. For example, mastery can successfully be improved with intervention (van der Klink et al., 2001; van der Zanden et al., 2012). Future studies can focus on subgroup replication and validation, but also on the development of interventions for those autistic adults who could benefit from extra support.

### Supplementary Information

Below is the link to the electronic supplementary material.

Supplementary file1 (DOCX 420 KB)

## Data Availability

Because of the nature of this research, participant’s data is not publicly available due to restrictions (i.e., data contain information that could compromise the privacy of research participants). Nonetheless, an anonymized person-to-person correlation matrix based on the original data set including autistic adults and comparison participants can be found at https://uvaauas.figshare.com/articles/dataset/A_community_detection_study_Two_replicated_distinct_subgroups_in_autistic_adults/21333633.

## Code Availability

The code for the community detection analysis applied to the original data set with data of autistic and comparison participants is uploaded at https://osf.io/5uyzd/.
