# Supplementary material for: Finding Similarities in Differences Between Autistic Adults: Two Replicated Subgroups
Source: J Autism Dev Disord. 2023 Jul 12;54(9):3449–66. doi: 10.1007/s10803-023-06042-2 (PMC11362251; doi:10.1007/s10803-023-06042-2)
Supplement: Supplementary file 1 — Supplementary file1 (DOCX 420 KB) [file 10803_2023_6042_MOESM1_ESM.docx]

Online Resource for:

**Finding similarities in differences between autistic adults: Two replicated subgroups**

**Table of content**

**1. Internal consistency for measures based on autism group in replication data**

**2. Missing data in original and replication data**

**3. Cluster variable correlation matrix based on original data of autistic and non-autistic adults**

**4. Distribution of scores on cluster variables across diagnostic groups for replication data**

**5. Profile plot for Autism and COMP subgroups formed on original data**

**6. Descriptive statistics for each of the three autism subgroups formed on replication data**

**7. Scores on external validation measures for the two replicated autism subgroups formed on original data**

**8. Scores on external validation measures for each of the three autism subgroups formed on replication data**

**1. Internal consistency for measures based on autism group in replication data**

sTable 1

*Internal consistency (Cronbach’s α) for the measures included as cluster variables, based on the autism group in the replication data.*

| **Measure^a^** | **Cronbach’s α** |
| --- | --- |
| AQ total | 0.85 |
| - Social skills | 0.72 |
| - Attention switching | 0.66 |
| - Attention to detail | 0.66 |
| - Communication | 0.66 |
| - Imagination | 0.59 |
| Sensory sensitivity | 0.62 |
| Mastery | 0.80 |
| Worry | 0.88 |
| Emotional support | 0.86 |
| Positive affect | 0.86 |
| Negative affect | 0.90 |

*Note.* ^a^ Cronbach’s alpha was not calculated for education, negative life events and physical activity, as these instruments are better described as formative measures rather than reflective of a latent trait.

**2. Missing data**

In the original data set there was 4.38% of missing data in total (i.e., 128 missing values out of 2925). In the replication data set, there was 1.96% of missing data in total (i.e., 201 missing values out of 10272). The percentage of missing data per cluster variable can be found in sTable 2.

On the item level, a maximum of 10% of missing data was recoded to the median of the participant’s other responses on this specific questionnaire. Afterwards, sum scores were calculated that resulted in scores on 14 cluster variables per participant. At the instrument level, we only included participants with no more than one missing value (i.e., at least 13 non-missing values on 14 cluster variables), and no imputation was performed.

After imputation of missing data at item level, and removal of cases who still had more than one missing value on instrument level, there were 172 cases in the original data set (i.e., 23 were excluded, 12%). Of these cases, there were 23 cases who still had a missing value on one of the cluster variables: four on education, one on imagination, one on sensory sensitivity, seven on emotional support, eight on physical activity, one on negative affect, and one on negative life events.

After imputation of missing data at item level, and removal of cases who still had more than one missing value on instrument level, there were 548 cases in the replication data set (i.e., 32 were excluded, 6%). Of these cases, there were 52 who still had a missing value on one of the cluster variables: five on education, five on sensory sensitivity, one on mastery, one on worry, 19 on emotional support, 14 on physical activity and seven on negative life events.”

sTable 2

*Number and percentages of missing data for each of the cluster variables in the original and replication data.*

| **Cluster variable** | **Data set** | |
| --- | --- | --- |
|  | Original | Replication |
|  | Percentage of missing values (%) | |
| Education | 2.05 | 1.25 |
| AQ social skill | 1.03 | 0.62 |
| AQ attention switching | 1.03 | 0.62 |
| AQ attention to detail | 1.54 | 0.62 |
| AQ communication | 1.54 | 0.62 |
| AQ imagination | 1.54 | 0.78 |
| Sensory sensitivity | 1.54 | 1.09 |
| Mastery | 0.51 | 0.62 |
| Worry | 0.51 | 0.47 |
| Emotional support | 13.33 | 3.58 |
| Physical activity | 13.33 | 6.70 |
| Positive affect | 8.71 | 4.36 |
| Negative affect | 9.23 | 4.36 |
| Negative life events | 9.74 | 5.60 |

**3. Cluster variable correlation matrix based on training data of autistic and non-autistic adults**

sTable 3
*Correlation matrix of cluster variables based on training data of autistic and non-autistic adults*

|  | **Edu** | **Soc** | **AttS** | **AttD** | **Com** | **Imag** | **Sens** | **Mas** | **Wor** | **Sup** | **Phys** | **NAff** | **PAff** | **Nlife** |
| --- | --- | --- | --- | --- | --- | --- | --- | --- | --- | --- | --- | --- | --- | --- |
| **Edu** | 1.00 |  |  |  |  |  |  |  |  |  |  |  |  |  |
| **Soc** | -0.16 | 1.00 |  |  |  |  |  |  |  |  |  |  |  |  |
| **AttS** | -0.17 | 0.78 | 1.00 |  |  |  |  |  |  |  |  |  |  |  |
| **AttD** | -0.05 | 0.47 | 0.55 | 1.00 |  |  |  |  |  |  |  |  |  |  |
| **Com** | -0.18 | 0.81 | 0.70 | 0.52 | 1.00 |  |  |  |  |  |  |  |  |  |
| **Imag** | -0.10 | 0.59 | 0.66 | 0.45 | 0.60 | 1.00 |  |  |  |  |  |  |  |  |
| **Sens** | -0.30 | 0.48 | 0.54 | 0.46 | 0.52 | 0.37 | 1.00 |  |  |  |  |  |  |  |
| **Mas** | 0.09 | -0.65 | -0.66 | -0.45 | -0.62 | -0.49 | -0.50 | 1.00 |  |  |  |  |  |  |
| **Wor** | -0.06 | 0.45 | 0.48 | 0.34 | 0.48 | 0.26 | 0.46 | -0.68 | 1.00 |  |  |  |  |  |
| **Sup** | 0.13 | -0.33 | -0.31 | -0.14 | -0.25 | -0.25 | -0.15 | 0.29 | -0.22 | 1.00 |  |  |  |  |
| **Phys** | 0.01 | -0.17 | -0.24 | -0.15 | -0.11 | -0.23 | -0.08 | 0.13 | -0.10 | 0.14 | 1.00 |  |  |  |
| **PAff** | 0.17 | -0.48 | -0.50 | -0.15 | -0.36 | -0.37 | -0.21 | 0.54 | -0.36 | 0.36 | 0.28 | 1.00 |  |  |
| **NAff** | 0.01 | 0.42 | 0.49 | 0.37 | 0.38 | 0.37 | 0.39 | -0.63 | 0.68 | -0.19 | -0.17 | -0.28 | 1.00 |  |
| **Nlife** | 0.02 | -0.05 | 0.04 | 0.18 | -0.03 | -0.01 | 0.02 | -0.04 | 0.19 | -0.02 | -0.01 | <-0.01 | 0.21 | 1.00 |

*Note.* Edu = education, Soc = social skills, AttS = attention switching, AttD = attention to detail, Com = communication, Imag = imagination, Sens = sensory sensitivity, Mas = mastery, Wor = worry, Sup = emotional support, Phys = physical activity, PAff = positive affect, NAff = negative affect, NLife = negative life events.

**4. Distribution of scores on cluster variables across diagnostic groups for replication data**

**
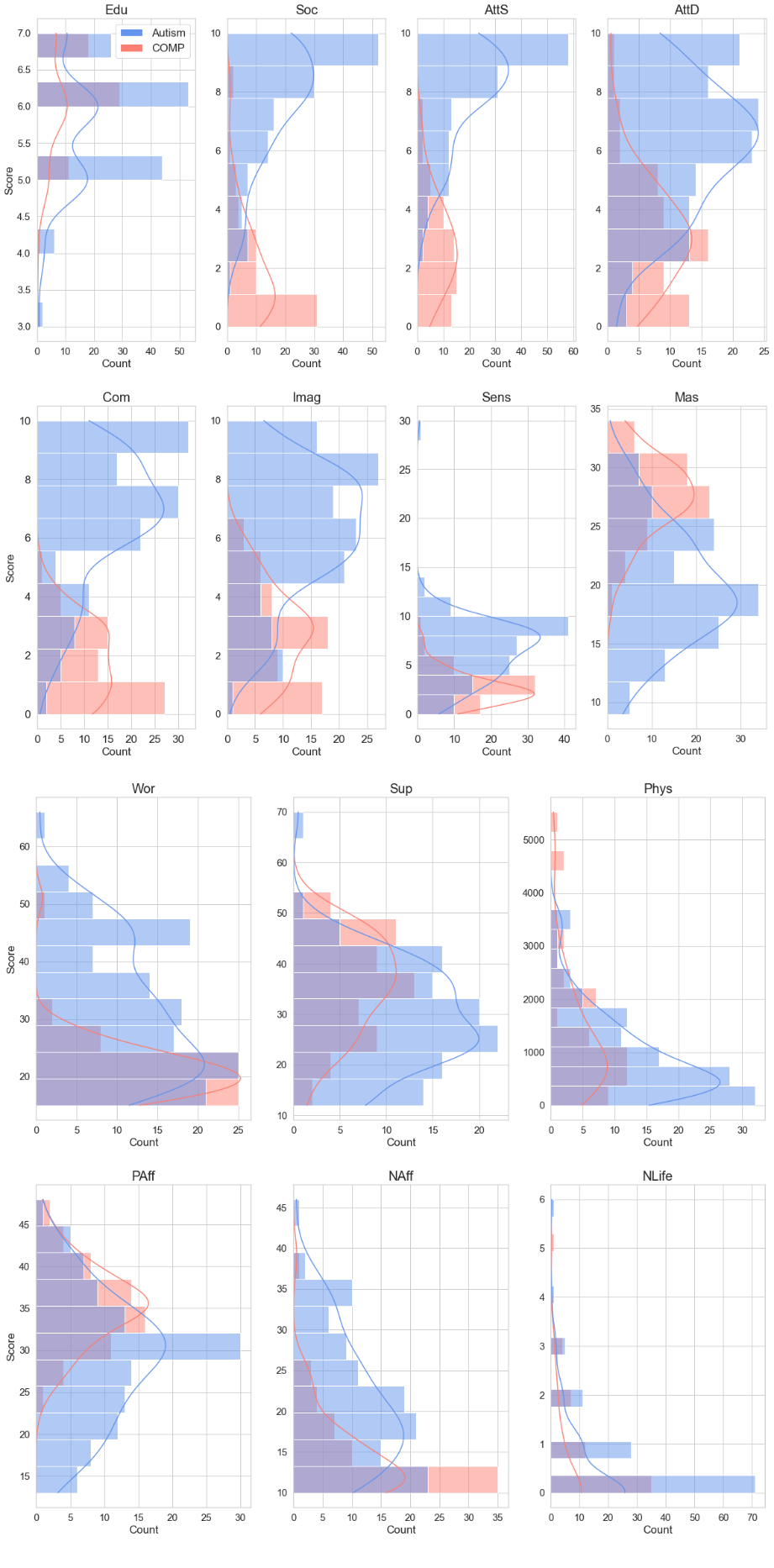
**

*sFigure 1.* Distribution of scores on cluster variables across diagnostic groups (i.e., Autism and COMP) for replication data. *Note.* COMP = comparison, Edu = education, Soc = social skills, AttS = attention switching, AttD = attention to detail, Com = communication, Imag = imagination, Sens = sensory sensitivity, Mas = mastery, Wor = worry, Sup = emotional support, Phys = physical activity, PAff = positive affect, NAff = negative affect, NLife = negative life events.

**5. Profile plot for Autism and COMP subgroups formed on original data**

**
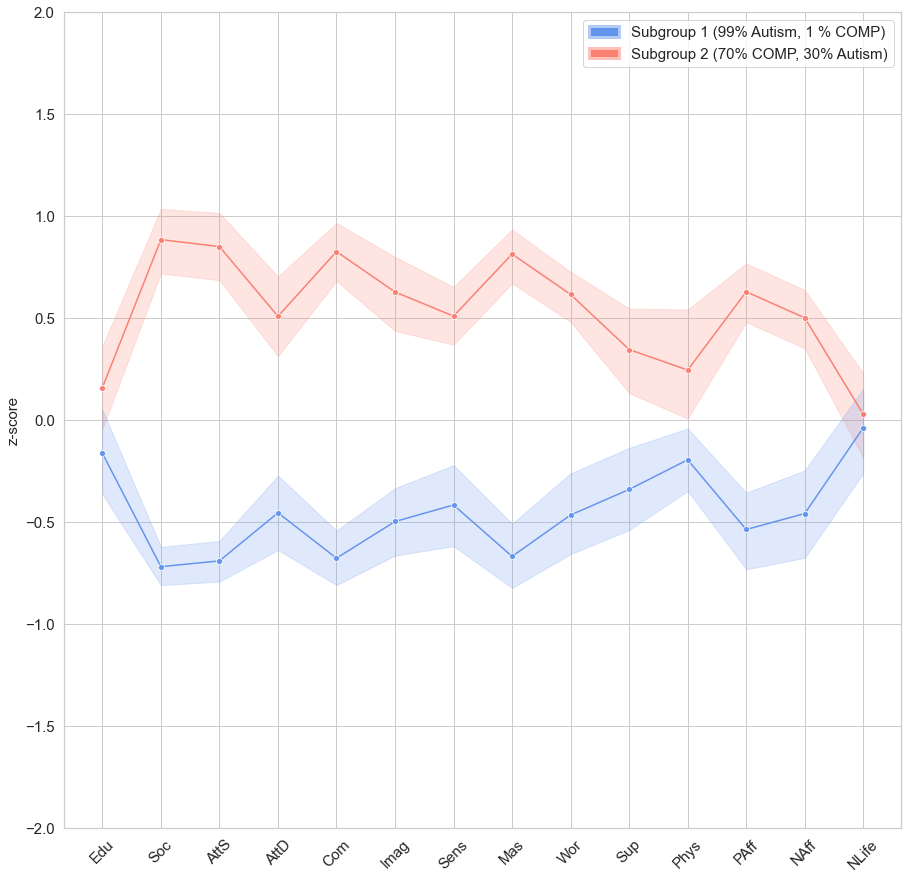
**

*sFigure 2.* Subgroup profiles based on data from the Autism and COMP groups for each of the two community detection-based subgroups formed on replication data.

*Note.* COMP = comparison, Edu = education, Soc = social skills, AttS = attention switching, AttD = attention to detail, Com = communication, Imag = imagination, Sens = sensory sensitivity, Mas = mastery, Wor = worry, Sup = emotional support, Phys = physical activity, PAff = positive affect, NAff = negative affect, NLife = negative life events. Higher *z*-scores represent higher scores on Edu, Soc, AttD, AttS, Com, Imag, Mas, Sup, Phys, PAff. Higher *z*-scores represent better scores on Sens, Wor, NAff, NLife (less sensitivity, less worrying, less negative affect, fewer negative life events). Shaded area represents 95%-confidence interval.

**6.** **Descriptive statistics for each of the autism subgroups formed on replication data**

sTable 4

*Raw cluster variable scores and descriptive for each of the three autism subgroups formed on replication data (N=261).*

|  |  | **Subgroup** |  |  |  |  |  |
| --- | --- | --- | --- | --- | --- | --- | --- |
|  | HighGr | LowGr | Rest |  |  |  |  |
| Variable | N = 124 | N = 130 | N = 7^d^ | test statistic(*df*) | HighGr vs. LowGr (*Z)* | HighGr vs. Rest^d^ (*Z)* | LowGr vs. Rest^d^ (*Z)* |
| **Cluster variables** |  |  |  |  |  |  |  |
| Education | 5.93 (0.91) | 6.00 (0.79) | 6.71 (0.49) | *F*(2, 255) = 2.87 | -0.39^a^ | -2.47*^a^ | -2.35*^a^ |
| AQ social skill | 6.43 (2.45) | 8.61 (1.36) | 8.29 (1.38) | *F*(2, 258) = 39.77*** | -7.71***^a^ | -1.88^a^ | 0.62 |
| AQ attention switching | 6.92 (2.23) | 8.72 (1.34) | 8.57 (0.79) | *F*(2, 258) = 31.93*** | -6.99***^a^ | -1.77^a^ | 0.49 |
| AQ attention to detail | 6.40 (2.41) | 6.95 (2.04) | 6.29 (1.89) | *F*(2, 258) = 2.06 | -1.88^a^ | 0.24 | 0.85 |
| AQ communication | 5.91 (2.27) | 7.55 (1.93) | 8.00 (1.41) | *F*(2, 258) = 20.69*** | -5.76***^a^ | -2.42*^a^ | -0.56^a^ |
| AQ imagination | 4.98 (2.02) | 6.84 (1.97) | 7.57 (1.27) | *F*(2, 258) = 30.24*** | -6.86***^a^ | -3.16*^a^ | -0.94^a^ |
| Sensory sensitivity | 6.53 (2.76) | 7.79 (2.27) | 6.00 (2.08) | *F*(2, 253) = 8.62*** | -3.79*^a^ | 0.70 | 1.94 |
| Mastery | 23.21 (4.30) | 16.50 (3.82) | 22.71 (5.06) | *F*(2, 257) = 86.96*** | 10.30*** | 0.33 | -3.01* |
| Worry | 28.99 (9.63) | 38.39 (11.33) | 36.57 (12.16) | *F*(2, 258) = 25.31*** | -6.67***^a^ | -1.74^a^ | 0.41 |
| Emotional support | 33.13 (10.72) | 24.75 (10.10) | 28.14 (8.97) | *F*(2, 251) = 20.13*** | 5.71*** | 0.91 | -0.96^a^ |
| Physical activity | 1314.80 (2028.13) | 908.81 (1022.04) | 635.33 (661.15) | *F*(2, 252) = 2.30 | 2.19* | 1.37 | 0.71 |
| Positive affect | 32.46 (6.43) | 25.04 (6.15) | 27.86 (6.39) | *F*(2, 258) = 44.26*** | 8.27*** | 1.66 | -1.01 |
| Negative affect | 18.44 (6.65) | 25.45 (8.36) | 15.00 (5.54) | *F*(2, 258) = 30.46*** | -6.74***^a^ | 1.22 | 3.40*** |
| Negative life events | 0.81 (1.07) | 0.73 (0.98) | 0.57 (0.53) | *F*(2, 255) = 0.36 | 0.49 | 0.16 | 0.01 |
| **Descriptive variables** |  |  |  |  |  |  |  |
| Age *M*(*SD*), range | 50.61 (13.76), 30-81 | 51.34 (11.58), 30-84 | 60.43 (10.01), 47-72 | *F*(2, 258) = 2.01 | -0.51^a^ | -2.04^a^ | -1.88^a^ |
| Biological sex^b^ |  |  |  | χ^2^(4) = 0.51 |  |  |  |
| % male | 53 | 44 | 57 |  |  |  |  |
| % female | 47 | 55 | 43 |  |  |  |  |
| IQ score^c^ | 116.70 (17,15) | 113.44 (15.25) | 120.50 (7.78) | *F*(2,93)=0.60 | 1.41 | -0.19 | -0.60 |

*Note.* HighGr = Feelings of high grip. LowGr = Feelings of low grip*.** *p* < 0.05, ** *p* < 0.01, *** *p* < 0.001. ^a^ Negative ­*z* test statistics indicate that the first mentioned group scores lower than the second group in the comparison. ^b^ The remaining percentage was classified as “other”. ^c^ Sample size is lower for this variable because data are only available for participants who completed the interview. ^d^ Please note that the group sizes were severely unbalanced in the comparisons that involved the Rest-subgroup (Stevens, 1996). Therefore, these results should not be used to draw conclusions regarding the Rest-subgroup. The results were included because all group comparisons were preregistered.

**7. Scores on external validation measures for the two replicated autism subgroups formed on original data**

sTable 5

*Scores for external validation measures for the two replicated autism subgroups formed on original data (N=97).*

|  | Subgroup | |  |  |
| --- | --- | --- | --- | --- |
|  | HighGr  N=49 (51%) | LowGr  N=48 (49%) |  |  |
| **Variable** | ***M (SD);* range** | ***M (SD);* range** | **Test statistic** | **Effect  size (d)** |
| Cognitive difficulties | 42.9(11.5); 21-65 | 52.4(15.1); 18-86 | *t*(95)=3.5* | -0.71 |
| SCL-90 total score | 135.9(26.7); 97-211 | 204.7(45.9); 124-303 |  | -1.83 |
| SCL-90 anxiety | 14.1(4.05); 10-15 | 22.0(7.9); 11-43 | *t*(69.7)=-6.2* | -1.27 |
| SCL-90 agoraphobia | 9.4(3.6); 7-25 | 13.3(3.9); 7-24 | *t*(95)=-5.1* | -1.04 |
| SCL-90 depression | 25.0(7.8); 16-56 | 40.8(11.5); 18-63 | *t*(82.6)=-7.9* | -1.61 |
| SCL-90 somatization | 17.7(4.5); 12-28 | 23.2(7.2); 12-40 | *t*(78.3)=-4.5* | -0.92 |
| SCL-90 cognitive performance deficits | 17.0(4.3); 9-30 | 24.4(5.9); 13-38 | *t*(86.0)=-7.1 | -1.46 |
| SCL-90 interpersonal sensitivity | 26.4(6.0); 18-43 | 42.9(11.5); 24-73 | *t*(70.6)=-8.8* | -1.81 |
| SCL-90 hostility | 8.1(2.3); 6-16 | 10.8(4.2); 6-22 | *t*(73.0)=-3.9* | -0.80 |
| SCL-90 sleep difficulties | 5.6(2.1); 3-11 | 8.1(3.3); 3-15 | *t*(80.1)=-4.4* | -0.89 |
| SCL-90 rest | 12.7(3.6); 9-24 | 19.0(5.9); 9-31 | *t*(77.1)=-6.5* | -1.75 |
| QoL Physical health | 14.8(2.5); 7-19 | 12.3(2.3); 7-19 | *t*(95)=4.9* | 0.99 |
| QoL Psychological | 14.1(2.3); 9-19 | 10.2(2.2); 6-17 | *t*(95)=8.6* | 1.74 |
| QoL Social relationships | 13.4(2.9); 7-19 | 10.6(3.2); 4-16 | *t*(95)=4.6* | 0.93 |
| QoL Environment | 16.3(2.2); 11-20 | 13.8(2.4); 10-19 | *t*(95)=5.5* | 1.12 |
| **Multivariate analyses** |  |  |  |  |
| SCL-90 |  |  | *F*(9,87)=10.1* |  |
| QoL |  |  | *F*(4,92)=19.1* |  |

*Note.* HighGr = Feelings of high grip. LowGr = Feelings of low grip. SCL-90 = Symptom Checklist. QoL = World Health Organization Quality of Life Questionnaire-BREF.

* *p* < 0.005.

**8. Scores on external validation measures for each of the three autism subgroups formed on replication data**

sTable 6

*Scores for external validation measures for each of the three autism subgroups formed on replication data (N=261).*

|  | **Subgroup** | | |  |  | |  |  | | | | |
| --- | --- | --- | --- | --- | --- | --- | --- | --- | --- | --- | --- | --- |
|  | HighGr | LowGr | Rest^c^ |  | HighGr vs. LowGr | HighGr vs.  Rest^c^ | | | LowGr  vs.  Rest^c^ | | |  |
| Variable | *M*(*SD*) | *M*(*SD*) | *M*(*SD*) | *F*(*df*) |  |  | | |  | | |  |
| Cognitive failures | 43.087 (14.84) | 51. 81 (13.83) | 50.57 (13.49) | *F*(2, 258) = 9.88* | -4.24*^b^ | -1.16^b^ | | | 0.21 | | |  |
| SCL-90 total score | 149.37 (40.97) | 199.31 (52.45) | 153.29 (33.86) | *F*(2, 255) = 36.28* | -8.05*^b^ | -0.29^b^ | | | 2.33 | | |  |
| SCL-90 anxiety | 15.93 (5.78) | 21.58 (7.74) | 15.86 (6.33) | *F*(2, 257) = 22.14* | -6.54*^b^ | 0.11 | | | 2.24 | | |  |
| SCL-90 agoraphobia | 9.29 (3.10) | 12.85 (5.05) | 11.29 (4.79) | *F*(2, 257) = 22.38* | -6.83*^b^ | -1.16^b^ | | | 1.05 | | |  |
| SCL-90 depression | 27.98 (9.06) | 40.56 (13.08) | 27.29 (7.20) | *F*(2, 257) = 41.08* | -7.93*^b^ | -0.03^b^ | | | 2.54 | | |  |
| SCL-90 somatization | 19.21 (6.62) | 22.52 (7.69) | 16.57 (3.87) | *F*(2, 255) = 7.99* | -3.99*^b^ | 1.05 | | | 2.35 | | |  |
| SCL-90 cognitive performance deficits | 18.00 (6.21) | 23.55 (6.78) | 19.00 (5.47) | *F*(2, 257) = 23.35* | -6.76*^b^ | -0.32^b^ | | | 1.86 | | |  |
| SCL-90 interpersonal sensitivity | 29.96 (10.00) | 41.12 (12.97) | 32.57 (7.55) | *F*(2, 256) = 29.74* | -7.31*^b^ | -0.80^b^ | | | 1.56 | | |  |
| SCL-90 hostility | 8.67 (3.34) | 10.38 (4.00) | 10.00 (3.87) | *F*(2, 257) = 6.81* | -4.44*^b^ | -1.08^b^ | | | 0.36 | | |  |
| SCL-90 sleep difficulties | 6.55 (2.90) | 8.47 (3.54) | 6.57 (2.07) | *F*(2, 257) = 11.58* | -4.37*^b^ | -0.27^b^ | | | 1.14 | | |  |
| SCL-90 not included in any specific factor | 13.79 (4.67) | 17.70 (5.48) | 14.14 (3.98) | *F*(2, 257) = 19.12* | -6.37*^b^ | -0.38^b^ | | | 1.69 | | |  |
| QoL Physical health | 14.28 (2.62) | 12.09 (2.56) | 12.71 (2.36) | *F*(2, 256) = 22.69* | 6.26* | | 1.39 | | | -0.61^b^ |  |  |
| QoL Psychological | 13.43 (2.29) | 10.75 (2.43) | 12.00 (2.24) | *F*(2, 257) = 40.60* | 8.01* | | 1.32 | | | -1.27^b^ |  |  |
| QoL Social relationships | 12.88 (2.80) | 10.68 (3.15) | 10.00 (2.00) | *F*(2, 258) = 18.62* | 5.40* | | 2.45 | | | 0.71 |  |  |
| QoL Environment | 15.83 (2.09) | 14.05 (2.42) | 13.86 (1.35) | *F*(2, 257) = 20.50 | 5.68* | | 2.38 | | | 0.54 |  |  |
| **Multivariate analyses** |  |  |  |  |  | |  | | |  |  |  |
| QoL |  |  |  | *F*(8, 504) = 11.15*^a^ |  | |  | | |  |  |  |
| SCL-90 |  |  |  | *F*(18, 494) = 5.45*^a^ |  | |  | | |  |  |  |

*Note.* HighGr = Feelings of high grip. LowGr = Feelings of low grip. SCL-90 = Symptom Checklist. QoL = World Health Organization Quality of Life Questionnaire-BREF.
* *p* < 0.005. ^a^ not corrected for multiple testing (i.e., *p* < 0.05). ^b^ Negative ­*z* test statistics indicate that the first mentioned group scores lower than the second group in the comparison. ^c^ Please note that the group sizes were severely unbalanced in the comparisons that involved the Rest-subgroup (Stevens, 1996). Therefore, these results should not be used to draw conclusions regarding the Rest-subgroup. The results were included because all group comparisons were preregistered.
